# Impact of the rural population aging on land ecological security

**DOI:** 10.3389/fpubh.2026.1694483

**Published:** 2026-06-12

**Authors:** Huishuang Jin, Yixuan Zhu

**Affiliations:** 1 School of Economics, Hunan Agricultural University, Changsha, China; 2 School of Business, Hunan Institute of Technology, Hengyang, Hunan, China

**Keywords:** aging, agricultural factor allocation, food, hunger eradication, land ecological security

## Abstract

The trend of demographic change is irreversible, and the aging of the rural population has become a significant factor undermining the foundations of food security. The relationship between this phenomenon and land ecosystems has not yet been thoroughly explored. To address this research gap, this study constructs an analytical framework of ‘aging—factor allocation—ecological security' to empirically examine the impact of rural population aging on land ecological security and its underlying mechanisms. The results indicate that aging exerts a significant negative impact on land ecological security; however, within the pathway through which aging influence land ecological security, aging promotes land ecological security by facilitating the adoption of agricultural production services, increasing land transfers, expanding large-scale farming operations, and adjusting crop patterns. The results of the heterogeneity analysis indicate that the impact of rural population aging on land ecological security varies. The negative impact of aging on land ecological security is more pronounced in western regions, areas with rugged terrain, among those with a medium level of education, and in sample groups not experiencing a low birth rate. This finding provides a rationale for policy interventions, suggesting that increasing inputs into productive agricultural services, promoting land transfer and large-scale operations, and increasing subsidies for planting food may be important pathways to improving land ecology and increasing food security.

## 1 Introduction

The world is currently facing an escalating food security crisis. In recent years, this crisis has been exacerbated by extreme climate change, geopolitical conflicts, biofuel production, population growth, and global economic downturns. Since the onset of the COVID-19 pandemic and the Russian-Ukrainian conflict, food prices have surged, and the achievement of zero hunger has become increasingly uncertain (1). According to the Food and Agriculture Organization of the United Nations (FAO), approximately 733 million people globally will suffer from hunger in 2023, and up to 99.1 million individuals are expected to be displaced by the food crisis in 2024, more than double the figure from 2013. Compounding this issue is the historically insufficient understanding of land ecological security and its importance to food security, leading to irrational land use practices worldwide (2). The misuse of agrochemicals, plastic packaging residues, and widespread land abandonment remain persistent problems.

As the most populous country in the world, China faces a particularly acute threat to food security. To achieve high agricultural yields, the country relies heavily on agrochemical inputs, which have become a “necessary evil”. Fertilization alone accounts for nearly 40% of China's increase in grain production, and the amount of fertilizer applied per unit area is far higher than the global average. Meanwhile, industrialization and urbanization have led to insufficient investment in the conservation of arable land, accelerating soil degradation and pollution. Without effective mitigation, land degradation and soil pollution will continue to threaten national food security. In this context, land ecological security has become an implicit yet crucial safeguard for food security. Addressing challenges to land ecological security is, therefore, essential.

Currently, academic research on land ecological security has gradually expanded from single-dimensional approaches such as evaluation, early warning, and evolutionary analysis to exploring spatial interconnectivity. Scholars have conducted research at various spatial scales, including river basins (3), regions (4), cities (5), and counties (6). Research has explored perspectives such as ecological efficiency (7–9), land abandonment (1), and ecological security. Influencing factors encompass agricultural policies (10), credit (11), green finance, urban land expansion (12), waste discharge from polluting enterprises (13), demographic transition (14–16), and famine experiences (17), among others.

Among the many contributing factors, population aging is increasingly becoming a focus of academic attention. However, existing research has not yet reached a consensus regarding the impact of aging on land ecological security. One view holds that population aging may have a positive impact on land ecosystems. Some studies have found that population aging has a significant positive impact on the green total factor productivity of agriculture, and that this beneficial effect can be further enhanced through the transfer of agricultural land (9). The underlying logic is that, constrained by health issues, reduced labor capacity and the relatively low returns from agriculture, older farmers may lack the incentive to expand their agricultural production, thereby reducing their use of fertilizers and pesticides in farming (18). Furthermore, the rapid development of agricultural mechanization has, to some extent, replaced manual labor, thereby not only reducing agriculture's reliance on physical labor but also minimizing post-harvest losses (19, 20). Another view holds that an aging population will hurt economic growth and the environment. With the large-scale migration of young and able-bodied workers from rural areas to cities, the aging of the remaining rural population is becoming increasingly pronounced, posing a threat to rural socio-economic development (21). As they grow older, older farmers find it difficult to cope with the physical demands of intensive agricultural work, and their productivity continues to decline (7, 8). Although agricultural services can replace some labor, when farmers lack the funds to purchase such services, this may also lead to farmland being left fallow (1). More importantly, older farmers may compensate for the labor shortage by increasing their expenditure on fertilizers, insecticides and herbicides (22). This strategy for the input of production factors will directly exacerbate soil pollution and ecological degradation.

In particular, there are two typical features of China's agricultural development: first, the aging of the rural working population is prominent. Aging is particularly pronounced in rural areas, where the old-age dependency ratio reached 30.94% in 2023, nearly double the urban ratio of 16.51%. Older adults have increasingly become the primary agricultural labor force; second, the smallholder business model with the family as the basic unit (19). While land allocation to households initially improved productivity and supported grain output growth, it also led to land fragmentation (23). In response, China has promoted large-scale farming through land transfers and property rights reform aimed at improving production efficiency.

In summary, existing research has examined the impact of population aging on land use from perspectives such as agricultural output, land abandonment and the use of agricultural chemicals; however, it has generally overlooked the impact of aging on the deeper ecological dimension of land ecological security, and there is a lack of systematic theoretical analysis and empirical testing of the underlying transmission mechanisms. In fact, demographic shifts profoundly influence land-use patterns and intensity by driving the reallocation of production factors, particularly land resources, thereby affecting land ecosystems.

This paper may contribute in the following three ways: First, by focusing on the impact of rural population aging on land ecological security, this study expands the scope of research in this field. Second, by constructing a theoretical analytical framework based on agricultural socialized services and land factor allocation, this study empirically tests the mediating roles of agricultural production services, land transfers, farm size, and crop structure, in an effort to clarify the channels through which population aging affects land ecological security. Third, by analyzing whether the impact of rural population aging on land ecological security varies according to factors such as economic development levels and educational attainment, this study contributes to a better understanding of the heterogeneous effects of population aging on land ecology.

## 2 Theories and hypotheses

The health of soil ecosystems is a key factor in addressing the food crisis. Against the backdrop of demographic and land-use changes, the key to analyzing the impact of an aging population on land ecological security lies in examining whether changes in production resulting from differences in factor allocation will have varying effects on land use.

To establish a rigorous theoretical analytical framework, this paper integrates the theories of factor allocation and ecological economics. According to factor allocation theory, farmers, acting as rational economic agents, maximize household income through productive services and the allocation of land resources, subject to labor supply constraints. In particular, the substitution effect of agricultural production services can help offset labor shortages. Land factor allocation helps improve land use efficiency. It mainly includes three aspects: land transfer, scale operation, and cropping structure. Faced with a steadily shrinking labor force, farmers may choose to transfer their land use rights. An aging workforce tends to favor grain crops, which are relatively easy to cultivate and manage and have a high degree of mechanization. This will lead to a shift in crop patterns toward a greater emphasis on grain production. Theories of ecological economics emphasize that the health of land ecosystems depends not only on natural resource endowments but is also influenced by the cumulative effects of long-term human land-use practices. Changes in the allocation of production factors—such as shifts toward large-scale farming and changes in production methods—may have far-reaching implications for the ecosystem services provided by land by altering the intensity of land management and the structure of factor inputs.

Therefore, constructs a framework to explore the effects of aging on land ecological security. It involves the direct effects of rural aging on the health of the land ecosystems, as well as the mediating effects of agricultural socialization services and land factor allocation. Among them, land factor allocation mainly includes land transfer, scale operation, and planting structure. Next, the study explores these 4 perspectives.

### 2.1 Direct impacts of rural population aging on land ecological security

Demographic change has been recognized as an adverse factor in environmental degradation (16), which directly affects land ecological security. From the perspective of labor supply, the shortage of labor triggered by the aging will directly result in the rough operation of agriculture or even the abandonment of farmland (4). This is because human labor capacity has an inverted U-shaped relationship with age. After the peak, the physical and human capital of older farmers deteriorates with age. Their household economic development capacity is weakened, and the labor force participation rate decreases, which affects the optimal farmland acreage (24). At the same time, farmland is facing a succession crisis due to the two forces of low birth rate and the younger generation of labor engaged in non-farm production (25). In order to maximize private gain, farmers will intensify land grabs. In addition, according to Maslow's hierarchy of needs (26), it can be seen that human consumption needs are organized according to a certain hierarchy, with different needs at different ages. Older people's digestive ability decreases, and their demand for food is relatively low. With a heavier labor burden, they considered it unnecessary to operate more arable land, consequently triggering farmland abandonment (1). Fallow land not only directly leads to a deterioration in soil aeration and permeability, but also allows weeds on the land to provide a breeding ground for pests and diseases, thereby hurting surrounding farmland and ecosystems on a larger scale. From the perspective of technology adoption, as seniors age, their social networks shrink, and combined with their lack of proficiency in digitization and the Internet, they have poor access to effective information. It is difficult for them to acquire new knowledge and techniques of agricultural production. In their production processes, the uptake of soil conservation techniques—including soil testing and fertilizer formulation, as well as biological pest and disease control—remains low. They overlook the long-term benefits of soil conservation. This lag in technological adoption and the lack of conservation practices not only hinder the sustainability of agricultural production but also further exacerbate the negative impacts on soil ecosystems (3). Therefore, this research proposes:

Hypothesis 1 (H1): Aging has a negative effect on the land ecological security.

### 2.2 Impacts of aging on land ecological security from the perspective of productive agricultural services

The theory of service-scale operations suggests that promoting farmers' participation in the division of labor can increase their productivity (27). Especially in China's situation of “big country, small farmers”, agricultural outsourcing services have become an important pathway for advancing agricultural modernization (28), effectively mitigating the adverse effects of aging on land ecological security. First, agricultural productive services exert significant labor substitution effects and can resolve the situation of insufficient labor capacity of the older population. Agricultural machinery replaces farmers' participation in agricultural production, which is conducive to suppressing farmland abandonment, promoting deep plowing and deep loosening of soil, and improving the water use efficiency (29), thus improving the ecological environment of farmland. Second, in the light of technology diffusion, the outsourcing by farmers to service organizations can break down the technological barriers and the barriers to technological innovation and investment in mitigation. Finally, from the point of view of economies of scale, service organizations aggregate farmers' demand for services. The larger the market volume of outsourcing demand, the stronger the negotiating power of service organizations. This helps them to purchase green factors of production from suppliers at a cost lower than the market price, inducing farmers to purchase ecologically based services, thereby upgrading the quality of the land. Therefore, this research proposes:

Hypothesis 2 (H2): Aging rural populations contribute to land ecological security by promoting productive agricultural services.

### 2.3 Impacts of aging on the land ecological security from a land transfer perspective

As rational economic agents, when faced with labor constraints, farmers will maximize their household income by adjusting the allocation of land resources (30). Land transfer plays a crucial intermediary role in the pathways through which population aging affects land ecological security. It is the core mechanism for the allocation of land resources. As left-behinds grow older, their health capital decreases, resulting in a “physical aging effect”. This leads to land being left unused. Under these constraints, older households can, by transferring part of their farmland to younger, able-bodied farmers, reduce both the wastage of land resources and carbon emissions intensity (19). Furthermore, older households are experienced in agricultural production and have unique advantages in crop rotation and the substitution of chemical fertilizers with farmyard manure. Land transfers can reduce the amount of farmland cultivated by older households, thereby increasing the efficiency with which they farm the remaining land. Compared to older households, younger agricultural operators have technological and talent advantages. This helps to enhance the application of advanced technologies, including smart farm machinery, precision agriculture, and digitalized production and marketing synergies. In addition, young farmers who have transferred their land tend to have stronger ecological awareness and market sensitivity, and they give priority to the premium price of green products and the sustainable use of land. Therefore, this research proposes:

Hypothesis 3 (H3): Land transfer can decrease the detrimental impacts of aging rural populations on the health of land ecosystems.

### 2.4 Impacts of aging on the land ecological security from the perspective of land management scale

Land fragmentation can hinder the application of agricultural technology, resulting in the inability to implement uniform pro-environmental behaviors. Land transfer and large-scale operation have become the basic trend of modern agricultural development. It is also the main line of China's agricultural policy (31). Meanwhile, it is also an important means of promoting green production among farmers (32). An aging population is leading to a labor shortage. Faced with increasing constraints on household labor, farmers are increasingly inclined to transfer their land management rights to new types of agricultural operators, including family farms, cooperatives and large-scale growers. This transfer process provides a practical foundation for large-scale farming, thereby influencing the land ecosystem in three key ways. Firstly, the standardization of production management. These business entities can implement unified field management for large areas of farmland, including sowing, fertilizing, and spraying. This standardized approach helps to promote the transition of agricultural production toward specialization and standardization, thereby increasing land productivity. Secondly, the modernization of technical equipment. Compared with small-scale farmers operating on a scattered basis, farm owners are better placed to introduce modern, advanced technology and equipment, including unmanned plant protection aircraft, water-saving irrigation systems, and agricultural sensors, to enable the scientific application of fertilizers and medicines, reduce environmental pollution, and thus protect the land ecosystem. Thirdly, the optimisation of the human capital structure. Research indicates that large-scale farming is able to attract young and middle-aged people to enter the agricultural sector, thereby counteracting, to some extent, the negative effects of an aging population (33). The younger generation of agricultural workers is better educated and has greater access to information; with their progressive ideas, they will accelerate the process of agricultural modernization. Thus, the following research hypotheses are proposed:

Hypothesis 4 (H4): Aging promotes land ecological security by promoting large-scale operations.

### 2.5 Impact of aging on land ecological security from the perspective of cropping structure

Agricultural production is dominated by older age groups, which, combined with low levels of human capital, make it difficult for them to master new agricultural technologies. They rely mainly on experience to grow food crops with low labor requirements. Food crops are characterized by relative concentration and regularity in the production chain. For example, some labor inputs are required for sowing and harvesting, and field management is simpler for other growth stages. This mode of farming facilitates the aging labor force with limited physical strength to rationalize their time and complete the production of food. The technology of grain crops is more mature, and the rate of agricultural mechanization is higher, which is beneficial for the application of modern agricultural technology. In contrast, cash crops are less mechanized and require more labor and expertise inputs, which is more difficult for older farmers with declining physical strength and energy. In addition, Governments usually exercise macro-control over food prices, with low price volatility and relatively stable income expectations. Farmers do not have to worry about not being able to recover the costs invested in green production due to large price fluctuations. Thus, this study proposes the research hypotheses:

Hypothesis 5 (H5): Aging contributes to the land ecological security by contributing to the “grain-orientation” of cropping structures.

**Figure 1:**
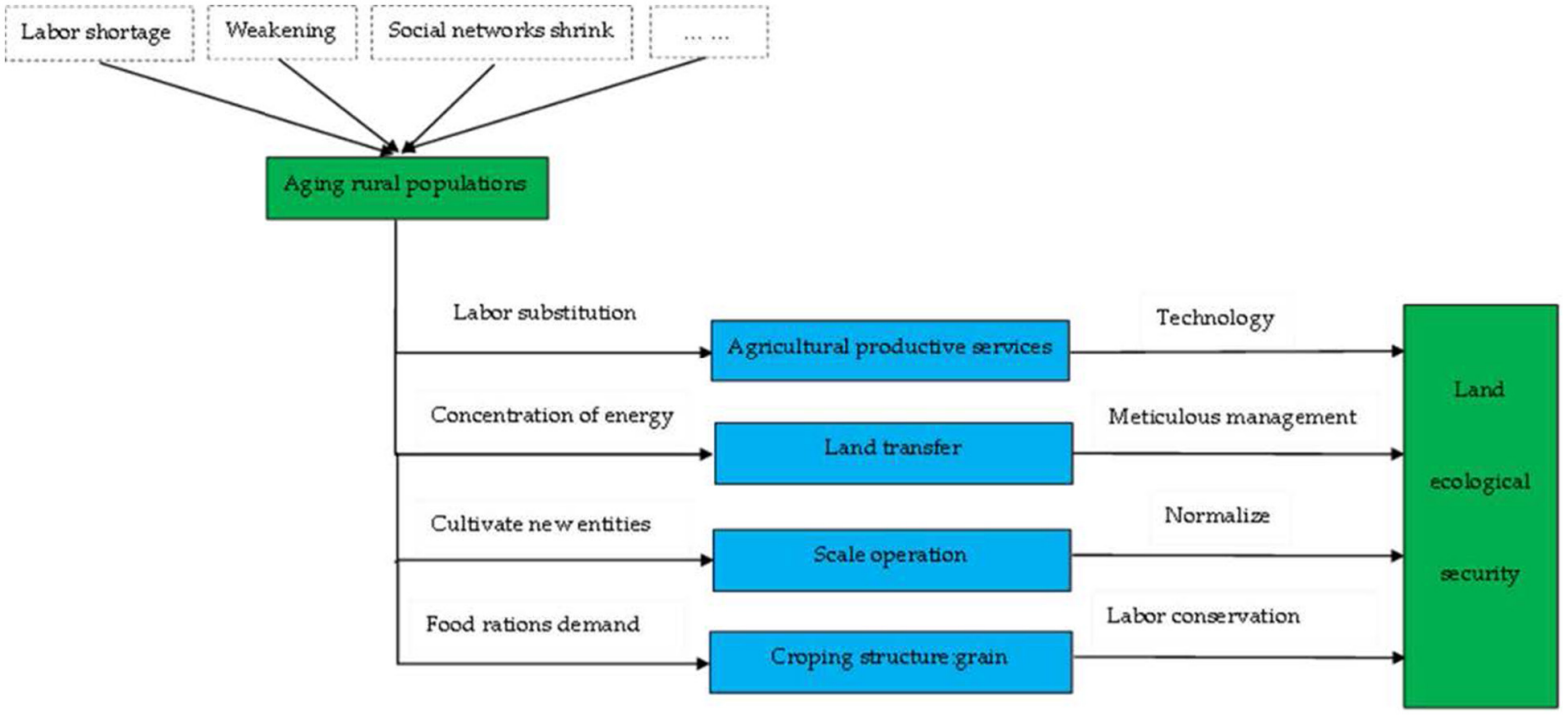
Conceptual framework of aging rural populations on land ecological security.

The analysis framework is depicted in Figure 1.

## 3 Materials and methods

### 3.1 Indicator construction

#### 3.1.1 Explained variables

The dependent variable in this study is land ecological security. Drawing on existing research, we designed an evaluation indicator system based on the PSR model, which decomposes land ecosystems into three dimensions: pressure, state, and response. See Table 1 for the specific evaluation criteria. This study employs the entropy-weighted TOPSIS method to calculate the land ecological security index.

**Table 1:** Indicator system for land ecological security evaluation.

Guideline level	Indicator level	Definition
Pressure	Industrial wastewater discharge	The quantity of industrial wastewater discharged
	Industrial SO_2_ emissions	Quantity of industrial SO_2_ emissions
	Disaster rate	Area affected by crops/area sown with crops
	Employment in the primary sector	Number of persons employed in the primary sector
	Fertilizer load per unit of cultivated area	Fertilizer use/cropland area
	Pesticide load per unit of cropland area	Pesticide use/cropland area
State	Water resources per unit of land	Total water resources/land area
	Population density	Permanent population/land area
	Urbanization rate	Urban population/total resident population
	Share of agricultural output	Agricultural output/GDP
	Cultivated land area per capita	Cultivated land area/total resident population
	Food production from arable land	Grain production/cultivated land area
	Crop sown area	Sown area of crops
Response	Investment in industrial pollution control	Investment in industrial pollution control as a share of GDP
	Daily urban wastewater treatment capacity	Daily sewage treatment capacity of the city
	Area of soil erosion control	Soil erosion control area/land area
	Cultivation rate of cropland	Cultivated land area/land area
	Effective irrigation of cropland	Effective irrigated area/cultivated area

The pressure dimension (P) reflects the pressure that human activities exert on the land's ecological environment. The discharge of industrial wastewater and exhaust gases directly affects soil systems, thereby altering soil pH levels. The discharge of industrial wastewater directly damages the ecological functions of farmland by contaminating soil and water resources. It also indirectly threatens food security and human health by affecting crop yields and the safety of agricultural products. Air pollutants are trace substances that are harmful to human health and the environment; they are released into the atmosphere through human activities or natural processes. When pollutant concentrations reach harmful levels, they can damage the health of ecosystems. Sulfur dioxide is one of the major pollutants. It can remain suspended in the air for long periods, not only interfering with plant photosynthesis but also contributing to the formation of acid rain in clouds. Furthermore, it alters the pH levels of farmland. Frequent agricultural disasters and worsening non-point source pollution place enormous pressure on ecosystems (34). The greater the area of crops affected by disasters, the greater the impact on agricultural output, and the more detrimental it is to the stable development of farmland. The use of chemical fertilizers is closely linked to grain yields. Excessive application of chemical fertilizers and pesticides can have adverse effects, including a reduction in beneficial soil microorganisms, disruption of soil structure, and soil compaction, all of which lead to a decline in soil fertility. When applied, chemical fertilizers and pesticides can also seep into water bodies, contaminating water sources. The number of people employed in the primary sector reflects changes in the agricultural workforce and has a significant impact on agriculture.

The status dimension (S) reflects the current state of economic development, demographic changes, and agricultural production. Water resources are a critical factor in agricultural production. Water shortages can lead to land degradation and reduced yields (12). This study uses water resources per unit of land as a measure. Factors such as population, industrial structure, and urbanization influence the potential for farmland conservation. The current state of the land, as reflected in grain production and crop planting area, indicates land use efficiency (2). Specifically, population density is a key indicator of changes in regional population distribution, and an increase in population density implies a rise in food demand. Urban development requires the use of large amounts of farmland. The faster the pace of urbanization, the more farmland is used. The development of the primary sector is closely linked to a region's arable land resources and the sustainable use of land. The higher the share of agricultural output in the economy, the more significant the positive role of agriculture in economic and social development. The amount of arable land per capita reflects the abundance of arable land resources. It is one of the key indicators used to assess the efficiency of land resource allocation and utilization. Grain yields on cultivated land directly reflect the productive potential of that land and the status of crop production. Grains are an essential staple food for the Chinese people. Increasing yields across large areas will help strengthen the capacity to ensure a stable grain supply. The area under crop cultivation serves as a measure of the intensity of arable land use in a given region.

The response dimension (R) reflects the strategies adopted by human society to address the degradation of the land's ecological environment. Pollution control is a key indicator of progress in restoring the natural environment (35). This study uses investment in industrial pollution control and urban wastewater treatment capacity as proxies. Soil erosion control, agricultural irrigation, and land reclamation reflect humanity's transformation of land systems. The larger the area covered by soil and water conservation efforts, the higher the level of protection for the region's soil and water resources. The growth of crops and the dissolution of fertilizers are inseparable from water resources. The construction of agricultural water conservancy facilities helps mitigate drought and promotes increased crop yields; the area under effective irrigation reflects this characteristic. The arable land reclamation rate quantifies the extent to which a region's arable land resources have been developed.

#### 3.1.2 Core explanatory variables

The core explanatory variable is aging rural population, which is calculated using the ratio of the number aged 65 and older in the countryside to the number of working-age individuals aged 15 to 64. It is closely connected with? the production and operation of a region, as well as to the supply of labor, and is one of the most important indicators of demographic change and socio-economic consequences from a demographic point of view.

#### 3.1.3 Mechanistic variables

The mediating variables in this study include agricultural productive services, land transfer, land scale operation, and cropping structure. In particular, with regard to the level of agricultural production services, this paper draws on existing research (36, 37) and uses the output value of the agricultural service sector to measure the level of agricultural production services. Specifically, gross value of agricultural services = (gross value of agriculture/gross value of agriculture, forestry, livestock and fisheries) × gross value of agriculture, forestry, livestock and fisheries services; The land transfer indicator is measured using a proxy for the total area of family contracted arable land transferred; The measurement of the land scale operation indicator refers to the World Bank's standard for the division of agricultural business entities, with 2 hectares the threshold, and defines farmers with an operation scale of 2 hectares or more as scale operators. Thus, this paper uses the number of farmers operating more than 2 hectares to measure the state of scale operation of land. The cropping structure is proxied by the share of area sown to food crops, which is also one of the most used proxy variables in existing studies and is measured more accurately.

#### 3.1.4 Control variables

Control variables include: (1) economic development, measured by per capita gross regional product; (2) farmers' income, measured by per capita disposable income of rural residents, which is logarithmized to reduce the absolute difference between the values; (3) agricultural mechanization, proxied by the total power of agricultural machinery; (4) investment in agriculture, proxied by the expenditures on agriculture, forestry, and water affairs; (5) medical care, measured by the number of village health rooms; (6) technological progress, measured by the number of agricultural patents granted based on IPC classification and logarithmized; (7) industrial structure, measured by the industrial structure index, which is specifically measured as follows: industrial structure upgrading = tertiary industry value-added ratio/secondary industry value-added ratio. The descriptive statistics of each variable are shown in Table 2.

**Table 2:** Results of descriptive statistics of variables.

Variable	Definition	Mean	SD
Land ecological security	Indices calculated using entropy weights—TOPSIS	0.279	0.095
Aging	Rural population aged 65 and over/rural population of working age	0.188	0.076
Land transfer	Area of arable land transferred under a family contract	1.378	1.352
Scale management	Number of households operating more than 2 hectares	0.346	0.410
Croping structure	Area sown in food crops/area sown in crops	0.649	0.140
Agricultural productive services	Value of agricultural services	0.087	0.088
Economic development	GDP per capita	5.663	3.065
Farmers' income	Per capita disposable income of rural residents (in logarithms)	9.325	0.520
Mechanization	Total power of agricultural machinery	0.338	0.291
Investment in agriculture	Expenditures on agriculture, forestry, and water affairs	52.642	29.111
Medical care	Number of village health centers	2.087	1.693
Technological progress	Agricultural patents granted (in logarithms)	6.660	1.286
Industrial structure	Share of tertiary value-added/share of secondary value-added	1.215	0.704

### 3.2 Research methodology

#### 3.2.1 Entropy weight-TOPSIS

This study employs the entropy-based TOPSIS method to assess land ecological security. The entropy-weighted TOPSIS method is an improvement upon the traditional TOPSIS evaluation method. Its core principle is as follows: First, indicator weights are determined using the entropy method based on the objective information reflected by each evaluation indicator. Second, the TOPSIS method is used to measure the proximity of the evaluation indicators to the positive and negative ideal solutions. Finally, a ranking of relative performance is established, with higher scores indicating better evaluation results. In other words, the higher the calculated comprehensive land ecological security index value, the higher thelevel of land ecological security and the stronger the sustainable productive capacity of the cropland system. For specific calculation steps, refer to existing research findings (38, 39).

#### 3.2.2 Model setup

The study used two-way fixed effects models to construct the baseline regression:


$$\begin{array}{c} Y_{it}=\alpha_{0}+\alpha_{1}X_{it}+\alpha_{2}Z_{it}+v_{i}+\sigma_{t}+\varepsilon_{it} \end{array}$$ (1)

where *Y*_*it*_ is the level of land ecological security in province i in year t, *X*_*it*_ is the degree of rural population aging in region i in year t; *Z*_*it*_ is a series of control variables; *v*_*i*_and σ_*t*_ denote area fixed effects and time fixed effects, respectively; ε_*it*_ denotes a random perturbation term; α_0_ denotes a constant term, and α_1_and α_2_denote the estimated coefficients.

Further, a mediating effects model was constructed to test the mechanism of action between aging and land ecological security:


$$\begin{array}{c} M_{it}=\rho_{0}+\rho_{1}X_{it}++\rho_{2}Z_{it}+v_{i}+\sigma_{t}+\varepsilon_{it} \end{array}$$ (2)

In the formula, *M*_*it*_ is the mediating variable, including agricultural productive services, land transfer, scale operation, and cropping structure; ρ_1_ and ρ_2_ denote the estimated coefficients.

### 3.3 Data sources

Based on the indicator system constructed in the previous section, and considering the actual situation of land ecological security, data availability, and other factors, this paper selects the panel data of 30 provinces in China (excluding Tibet, Hong Kong, Macao and Taiwan) from 2009 to 2022 for empirical analysis. The data were mainly obtained from the China Rural Statistical Yearbook, China Population and Employment Statistical Yearbook, China Rural Management Statistical Yearbook, China Rural Policy and Reform Statistical Yearbook, provincial statistical yearbooks, emission source statistical yearbooks, water resources bulletins, the official website of the National Bureau of Statistics (NBS), and the EPS database for the corresponding years. In the collection and processing of data, linear interpolation was used to supplement individual missing data by speculation.

## 4 Results and discussion

### 4.1 Empirical findings of the baseline regression

Table 3 reports the results of the baseline regression of rural population aging on land ecological security. Columns (1)–(2) show random effects and fixed area models. Columns (3) show two-way fixed effects models. Column (4) incorporates control variables based on column (3). It shows that the aging takes a heavy toll on land ecological security. The estimated coefficient of rural population aging is negative at the 1% level.

**Table 3:** Baseline regression results.

Variables	(1)	(2)	(3)	(4)
Aging	−0.105^**^ (0.046)	−0.107^**^ (0.047)	−0.074^*^ (0.043)	−0.115^***^ (0.043)
Economic development				−0.000 (0.001)
Farmers' income				−0.081^***^ (0.023)
Mechanization				0.098^***^ (0.017)
Investment in agriculture				−0.001^***^ (0.000)
Medical care				0.024^**^ (0.011)
Technological progress				−0.007^***^ (0.002)
Industrial structure				−0.007 (0.006)
Constant	0.299^***^ (0.023)	0.299^***^ (0.009)	0.293^***^ (0.008)	1.052^***^ (0.209)
Area fixed	NO	YES	YES	YES
Time fixed	NO	NO	YES	YES
Observations	420	420	420	420
R-squared	0.019	0.019	0.961	0.968

^***^, ^**^, and ^*^ represent 1%, 5%, and 10% significance levels.

In terms of control variables, according to column (4), it was found that the level of farmers‘ income has a negative effect on land ecological security. The likely reason for this is that farmers are more dependent on non-farm employment for their income levels. As income levels rise, the opportunity cost of agricultural production increases. Farmers, to maximize their benefits, will resort to roughing it to save time involved in agricultural production, thus earning non-agricultural income. Agricultural investment significantly and negatively affects land ecosystem health, which is similar to the findings of an existing study (28). Agricultural technological advancement has a negative effect on land ecosystem health. This also confirms that not all agricultural technologies mentioned earlier are pro-environmental (40). For example, chemical fertilizers were invented in agricultural technology to improve soil fertility. However, over-reliance on chemical fertilizers has led to soil compaction, which is harmful to the ecosystem. The level of agricultural mechanization promotes land ecological security. The level of rural healthcare promotes land ecosystem health. The possible reason for this is that by spreading health knowledge and publicizing the dangers of pesticides and other agrochemical residues, health care workers in rural health offices invariably raise the farmers' awareness of green production, thus promoting soil health.

### 4.2 Robustness and endogeneity tests

(1) Replacing the explanatory variables

In this paper, principal component analysis was used to re-measure the level of land ecological security, and the regression results are shown in column (1) of Table 4. After replacing the measures of the explanatory variables, the estimated coefficients of the core explanatory variables are negative at 1% statistical level, and are not substantially different from the baseline regression results.

(2) Replacing the sample interval

**Table 4:** Robustness test and endogeneity treatment results.

Variables	(1)	(2)	(3)	(4)	(5)
	Replace the explanatory variables	Replace the sample interval	Consider extreme values	First stage	Second stage
Aging	−0.648^***^ (0.164)	−0.105^*^ (0.049)	−0.118^***^ (0.042)		−0.163^**^ (0.079)
IV				0.641^***^ (0.130)	
Control variables	YES	YES	YES	YES	YES
Area fixed	YES	YES	YES	YES	YES
Time fixed	YES	YES	YES	YES	YES
Kleibergen-Paap rk LM statistic					24.866
F-test				24.11	
Observations	420	378	420	390	390
R-squared	0.930	0.962	0.970	0.446	

^***^, ^**^, ^*^represent 1%, 5%, 10% significance levels.

Beijing, Tianjin, and Shanghai have the best economic base and a lower share of agriculture, and their inclusion in the model may lead to biased estimation results. Therefore, this paper re-estimates the model after excluding Beijing, Tianjin, and Shanghai from the overall sample, and the regression results are shown in column (2) of Table 4. Compared with the previous paper, the coefficient of rural population aging is still negative, and the main conclusion still holds.

(3) Considering the effect of extreme values

To avoid the effects of outliers and extreme values on the regression results, the variables were re-estimated after two-way shrinkage, and the regression results are shown in column (3) of Table 4. Rural population aging negatively affects the land ecological security index at the 1% statistical significance level, further indicating the reliability of the study's findings.

(4) Instrumental variable estimation considering endogeneity

Even though this paper controls for a range of control variables and conducts a number of robustness checks, there may still be endogeneity issues, such as omitted variables and measurement error. To address potential endogeneity issues, this paper draws on existing research to construct instrumental variables and estimate them using the 2SLS model. This is done by first using the intensity of family planning in 2000–2003 as an instrumental variable for aging, measured by the share of the number in the region who had practiced birth control in that year, in the number of women of childbearing age at the end of the year. Higher historical family planning intensity indicates more aging during the sample period of this paper and satisfies the correlation requirement. From an exclusionary perspective, it is difficult for family planning intensity as a population policy to have a direct impact on current land ecological security. Therefore, historical family planning intensity is a better instrumental variable for population aging. Second, since the intensity of family planning implementation is a variable that doesn't change over time, for the instrumental variable to satisfy the dynamics of the two-way characteristics of time and area, the aging with a lag of one period is used as the time-varying factor for this instrumental variable. Theoretically, it is difficult for land ecological security to affect rural demographics with a lag of 1 period, well avoiding the problem of reverse causation, and the results of the regressions are shown in columns (4) and (5) of Table 4. The results show that there is a positive correlation between instrumental variables and aging. The F statistic is 24.11, and the Kleibergen-Paap rk LM statistic is 24.866, indicating that instrumental variables are valid. The regression coefficient for rural population aging is significantly negative at the 1% statistical level, indicating that the conclusions of this paper still hold after endogeneity treatment.

In summary, the estimation results of the benchmark regression in this paper are robust.

### 4.3 Impact mechanism test

To verify the effect mechanism of aging on land ecological security, this includes four mediating variables, namely, agricultural productive services, land transfer, scale operation and cropping structure, into the analysis framework and constructs a mediating effect model for testing, and the results are shown in Table 5. Column (1) shows that the aging has a positive influence on agricultural productive services and is at 1% statistical significance. This suggests that the aging of rural populations drives the agricultural productive services, which in turn promotes land ecological security. Agricultural productive services play a mediating role. The likely reason is that the aging triggers a shortage of labor, forcing farmers to turn to specialized service organizations to inject new dynamics into agricultural production. Service organizations tend to adopt standardized and green technologies compared to individual farmers. This not only enables the service provider to receive financial subsidies, but also creates a positive social image for the enterprise.

**Table 5:** Results of the mediation effect test.

Variables	(1)	(2)	(3)	(4)
	Agricultural productive services	Land transfer	Scale management	Croping structure
Aging	0.298^***^ (0.080)	4.950^***^ (0.828)	0.437^***^ (0.147)	0.382^***^ (0.088)
Control variables	YES	YES	YES	YES
Area fixed	YES	YES	YES	YES
Time fixed	YES	YES	YES	YES
Observations	420	420	420	420
R-squared	0.895	0.939	0.978	0.965

^***^ represent 1% significance levels.

Column (2)–(4) shows that the aging has a positive influence on land transfer, large-scale operation, and “grain-oriented” cropping structure. It suggests that the three land use patterns mediate the effects of aging on land ecological security. In practice, farmers can promote sustainable agricultural development by adjusting the way land elements are allocated. By reconfiguring the organization, land transfer policy is beneficial to the flow of land to young business entities while addressing the labor constraints caused by aging, thereby promoting green production. Scale operators advocate the reduction of agricultural costs and the improvement of comparative returns through reduction techniques. Population aging will drive a shift toward grain-oriented crop patterns, which is consistent with the findings of previous studie (41). With high mechanization rates of the grain-growing structure and stable prices for agricultural products, farmers have more incentives to adopt sustainable green production methods, thereby promoting economic and ecological “double security”.

### 4.4 Heterogeneity analysis

In this paper, heterogeneity tests were conducted by grouping the data according to economic development, topographical relief, educational attainment and fertility rates. The estimation results are presented in Tables 6, 7.

**Table 6:** Results of the tests on economic development and topographical heterogeneity.

Variables	Level of economic development			Terrain undulation		
	(1) East	(2) Central	(3) West	(4) Small	(5) Medium	(6) Large
Aging	−0.026 (0.067)	0.085 (0.181)	−0.092^*^ (0.054)	−0.069 (0.066)	0.044 (0.073)	−0.142^**^ (0.058)
控制变量	YES	YES	YES	YES	YES	YES
Area fixed	YES	YES	YES	YES	YES	YES
Time fixed	YES	YES	YES	YES	YES	YES
Observations	154	140	126	266	84	70
R-squared	0.980	0.930	0.991	0.969	0.973	0.997

^**^, ^*^represent 5%, 10% significance levels.

**Table 7:** Test of heterogeneity in educational attainment and the declining birth rate.

Variables	Educational			Declining birth rate	
	(1) Low	(2) Medium	(3) High	(4) Yes	(5) No
Aging	−0.089 (0.084)	−0.243^**^ (0.116)	−0.058 (0.078)	0.060 (0.099)	−0.161^***^ (0.055)
Control variables	YES	YES	YES	YES	YES
Area fixed	YES	YES	YES	YES	YES
Time fixed	YES	YES	YES	YES	YES
Observations	136	138	139	107	313
R-squared	0.981	0.968	0.982	0.983	0.971

^***^, ^**^ represent 1%, 5% significance levels.

The results in columns (1) to (3) of Table 6 indicate that the impact of the aging rural population on land ecological security is not significant in the eastern and central regions, but is significantly negative in the western region. Possible reasons for this outcome include the relatively fragile ecological environment in the western regions, their relatively underdeveloped economy, and the significant outflow of the rural labor force. At the same time, the topography of the western regions is relatively complex; many modern agricultural technologies, particularly large-scale agricultural machinery, struggle to operate in hilly and mountainous terrain, and the land ecosystems are more sensitive to changes in population structure. The aging of the rural population has significantly reduced the land ecological security index.

The results in columns (4) to (6) of Table 6 show that, in areas with high topographic relief, the negative impact of population aging on land ecological security is significant, whereas it is not significant in the low and medium groups.

Possible reasons for this outcome include the fact that, in areas with rugged terrain, plots are generally small and the roads are rough, making it difficult for agricultural machinery to operate in such areas, which in turn leads to farmland being abandoned. On plots where crops are still grown, the uneven terrain makes it difficult to transport farmyard manure, so large quantities of chemical fertilizers have to be applied, leading to soil acidification. At the same time, farming on hilly terrain is prone to causing soil erosion.

The results in columns (1) to (3) of Table 7 indicate that the impact of rural population aging on land ecological security is significantly negative when the level of education is moderate, but not significant in the low and high education groups. Possible reasons include: groups with a moderate level of education may find themselves in a transitional phase between traditional and modern production methods; they have not yet fully mastered scientific land management techniques and are prone to being driven by short-term economic gains, leading to the overexploitation or mismanagement of land resources, which in turn has a significant negative impact on land ecological security.

As China's natural population growth rate enters negative territory, the declining birth rate may become another significant demographic shift. To examine the impact of the low birth rate on land ecological security, this paper introduces the variable of the rural child dependency ratio. In the study, a threshold of 20% is set: groups with a child dependency ratio higher than 20% are defined as the non-low-birth-rate sample group and assigned a value of 0; groups with a child dependency ratio lower than 20% are defined as the low-birth-rate sample group and assigned a value of 1. The results in columns (4) and (5) of Table 7 show that, in the low-birth-rate sample group, the impact of population aging on land ecological security is not significant. Conversely, in the non-low-birth-rate sample group, the impact of population aging on land ecological security is significantly negative. When the burden of supporting family members is particularly heavy, the older adults are forced to maintain land productivity through intensive farming and heavy use of pesticides. While this practice ensures the livelihood of multiple generations within the family, it exacerbates soil degradation and non-point source pollution. At the same time, regions with a high proportion of children are typically economically underdeveloped and lack non-agricultural vitality. In such regions, local governments provide limited financial support for ecological policies such as fallow rotation and straw return to the fields. Consequently, out of a desire to avoid risk, older adults are generally reluctant to adopt land conservation techniques.

### 4.5 Discussion

The results indicate that the rise in the rural older population will be detrimental to the sustainable development of agriculture. This demographic change negatively affects the health of land ecosystems. This conclusion demonstrates concordance with extant research findings in the field (14). The results also show that the aging promotes the development of productive agricultural services, echoing the findings of Zhang and Luo (17). In particular, rural China has always been a traditional rural society bound by “blood” and “geographic” ties, and multigenerational cohabitation is very common. The first generation of family members is predominantly an older age group, with a focus on agricultural production. The second and third generations are the main non-agricultural workers with complex social networks and higher levels of human capital. At once, China's labor mobility is mostly migratory, and its frequent cultural exchanges with towns and cities can convey new technologies and ideas to friends and relatives in the outflow areas, creating knowledge spillovers. In this scenario, intergenerational knowledge transfer facilitates the transition of production to mechanization and greening. In addition, the transformation of land use patterns in land transfer, large-scale management, and cultivation structure can mitigate the adverse effects of aging. This is consistent with the study of Huang et al. (19) that land transfer and large-scale management can mitigate the adverse effects of aging on carbon emissions. Moderate-scale expansion and agricultural transformation can achieve agricultural carbon reduction goals and promote land ecological security.

This study provides valuable insights for understanding rural population aging and land ecological security. The findings of this study apply to economies that are rapidly aging, dominated by smallholder farming, and where the market for agricultural social services is still developing. There are limitations to this study. Firstly, this study utilized provincial-level panel data from China and did not cover other countries or regions, which limits the spatial generalizability of the findings. Future research could attempt to integrate multi-source, cross-national data from organizations such as the Food and Agriculture Organization of the United Nations (FAO) and the World Bank to examine whether the impact of aging on land ecosystem security varies across countries with different levels of economic development, land tenure systems, and stages of demographic transition. Furthermore, the study will utilize more detailed spatial-level data (such as data at the prefecture-level city or county level in China) to more accurately measure the degree of aging and the status of land ecosystems, and to analyze the heterogeneity of impacts across different cultural and climatic contexts. Secondly, although this study examines the intermediary role played by agricultural production services, it does not specify the types of such services (such as tillage, harvesting, fertilization and pesticide application), nor does it quantify the quality of agricultural production services. This makes it impossible to accurately estimate the magnitude of the effect. In future, our research could incorporate micro-level survey data to distinguish in detail between different types of agricultural production services—such as mechanization services, technical services and agricultural input supply services—and examine their distinct mediating roles in the pathways through which population aging affects land ecological security. At the same time, we could develop a comprehensive evaluation framework based on factors such as service timeliness, technological suitability and farmer satisfaction, thereby further elucidating the role of service quality in the relationship between population aging and land systems. Thirdly, although this study analyses the impact of population aging on land ecological security, due to data limitations, it has not been possible to measure the extent of aging in specific terms (for example, the increasing prevalence of aging in age groups such as 65–70 and 70–75). As a result, the findings of this study do not fully reflect the heterogeneity among regions with varying degrees of aging. Future research could utilize data from multi-wave longitudinal surveys to further explore the varying impacts of different age groups within the older population on land ecological security, as well as the mechanisms underlying these effects. Finally, with the rapid development of digital technologies such as the Internet of Things, big data and artificial intelligence, and their deep penetration into the agricultural sector, the impact of digital technologies on land ecosystems is becoming increasingly apparent and has emerged as a significant factor that cannot be overlooked. Against this backdrop, future research will further examine the moderating role of digital technologies in the relationship between population aging and land ecological security from the perspectives of digital infrastructure, digital information systems and the digitalisation and intelligentisation of agriculture, thereby providing this study with more timely extensions and supplements.

## 5 Conclusions and suggestions

### 5.1 Conclusions

Based on the reality of demographic change and sustainable agricultural development, this study empirically examines the impact of aging on land ecological security using panel data in China. The study shown that: (1) Aging has a significant inhibitory effect on land ecological security, which still holds after a series of robustness and endogeneity tests. (2) Mechanism analysis shows that rural population aging will further promote land ecological security by influencing factor allocation, and that productive agricultural services, land transfer, large-scale operation, and cropping structure play a mediating role in the influencing mechanism. (3) Heterogeneity analyses found significant differences in the effects of rural population aging on land ecological security across four dimensions: level of economic development, topographic relief, education, and lesser childbearing. The negative effect of aging on land ecological security was more significant in the west and areas with high topographic relief, while the effect was relatively weak in the east, central, low relief, and medium areas; aging had a significant inhibitory effect on the medium-education group, while the effect was not significant on the low- and high-education groups; and the negative effect of aging on land ecological security was significant in the non-minority samples, but not in the minority sample group.

### 5.2 Suggestions

First, increase investment in productive agricultural services. Provide mechanized operation services for aging farmers through production trusteeship, substitute plowing and planting, and reduce the rate of abandonment of cropland. Increase investment in the construction of agricultural infrastructure, and actively carry out operations such as land leveling, water-saving irrigation, and repairing mechanized cultivation roads, to create conditions for the application of machinery. Strengthening the construction of the productive service, innovating the service model, promoting the combination of digitalization, intelligence, and production linkage trusteeship, and enhancing the ability of service organizations to link up with and bring farmers from both the supply and demand ends. At the same time, service organizations should strengthen the publicity of land protection knowledge and stimulate the transformation of production to scale, specialization, and greening through a combination of active publicity and demonstration leadership.

Secondly, the utilization pattern of land elements should be optimized. Improve the land transfer mechanism, implement standardized transfer contract models, and increase the duration of land transfer, thereby stabilizing land management rights. It is necessary to build regional land transfer platforms, dynamically supervise land transfers, boost the land transfer market, and create favorable opportunities? for large-scale operations. A database on the dynamics of the utilization of arable land should be established to monitor abandonment and changes in land use through satellite remote sensing. At the same time, it has increased financial investment in rural areas, promoted the construction of high-standard farmland, and subsidized large-scale farmers who operate in a contiguous area. The standards for grain-planting subsidies and land-use subsidies should be raised, to encourage capable older farmers to plant food crops that require less labor, effectively protect arable land, and curb the “non-grain-orientation” of arable land.

Third, optimize the regional industrial structure and accelerate the progress of industrial integration. In the production link with the help of digital technology to achieve precision agriculture, so as to promote the transformation of traditional to intelligent agriculture, and consolidate the foundation of agricultural modernization. Strengthening the traceability of the entire process of processing products and enhancing consumer trust in the quality and safety of products. Increase the comparative returns of agricultural operations by building brands for agricultural products. It is necessary to build an e-commerce sales system for products, shorten the agricultural industry chain of products from field to table, and promote the integration of one, two and three industries in agriculture, to attract some of the farmers who go out to work to return to their hometowns to start their own businesses.

## Data Availability

The datasets presented in this study can be found in online repositories. The names of the repository/repositories and accession number(s) can be found below: https://www.stats.gov.cn/.
